# Primary membranous nephropathy in two siblings with one combined with anti-glomerular basement membrane disease: a case report

**DOI:** 10.1186/s12882-023-03132-2

**Published:** 2023-06-22

**Authors:** Yan-jiao Cheng, Xiao-yu Jia, Hong-ru Cao, Xiao-yi Zhao, Xu-jie Zhou, Xiao-juan Yu, Rong Xu, Fu-de Zhou, Su-xia Wang, Zhao Cui, Ming-hui Zhao

**Affiliations:** 1 Renal Division, Peking University First Hospital, Beijing, 100034 PR China; 2 Institute of Nephrology, Peking University, Beijing, 100034 PR China; 3 Key Laboratory of Renal Disease, Ministry of Health of China, Beijing, 100034 PR China; 4 Key Laboratory of CKD Prevention and Treatment, Ministry of Education of China, Beijing, 100034 PR China; 5 Renal Division, Affiliated Hospital of Chifeng University, Chifeng, 024005 PR China; 6 Peking-Tsinghua Center for Life Sciences, Beijing, 100871 PR China

**Keywords:** Primary membranous nephropathy, Anti-PLA2R antibody, HLA, Anti-glomerular basement membrane disease, Case report

## Abstract

**Background:**

The phospholipase A2 receptor (PLA2R) associated with membranous nephropathy (MN) is an organ-specific autoimmune disease associated with *PLA2R* and human leukocyte antigen (*HLA*) genes. Familial PLA2R-related MN is rarely reported. The combination of anti-GBM disease and MN has been well documented, though the mechanism behind it remains unclear.

**Case presentation:**

We describe two siblings diagnosed with pathology-confirmed PLA2R-related MN 1 year apart. And one of the two siblings developed an anti-GBM disease. The high-resolution HLA typing showed identical alleles in both siblings, specifically heterozygotes of DRB1*15:01/*03:01.

**Conclusion:**

We describe a familial case of PLA2R-related MN supporting the role of genetic factors that HLA-DRB1*15:01 and DRB1*03:01 predispose patients in the development of PLA2R-related MN in the Han Chinese population. The combination of MN and anti-GBM disease may also partially be associated with the same susceptible HLA allele DRB1*15:01.

## Background

Primary membranous nephropathy (MN) is an organ-specific autoimmune disease characterized by the formation of subepithelial immune deposits, with the identification of underlying antigen, such as M-type phospholipase A2 receptor (PLA2R) [1], thrombospondin type-1 domain-containing 7A (THSD7A) [2], Exostosin 1/Exostosin 2 [3], and neural epidermal growth factor-like 1 (NELL-1) [4], etc. PLA2R-related MN accounted for 70–80% of primary MN [5]. Anti-glomerular basement membrane (GBM) disease is also an autoimmune disorder with target antigen as the noncollagenous (NC1) domain of α3 chain of type IV collagen [α3(IV)NC1] and manifests as crescentic nephritis with rapid progression of kidney dysfunction. The combination of anti-GBM disease and MN has been well documented [6, 7], though the mechanism behind it remains unclear.

Genetic susceptibility especially human leukocyte antigen (HLA) genes are essential for the mechanism of autoimmune diseases. There was evidence of associations between HLA class II and both primary MN and anti-GBM disease [8–12]. But the report of familial PLA2R-related MN was rare, as was anti-GBM following PLA2R-related MN. Here we presented two siblings with PLA2R-related MN and one of them developed the anti-GBM disease after MN, both of whom carrying risk alleles of HLA-DRB1.

## Case presentation

### Case 1

A 60-year-old female patient was admitted to our hospital with a complaint of proteinuria for 1 year in January 2021. She had hypertension, without other medical history or toxic or cigarette exposure. The blood pressure was 124/83 mmHg on admission, with no overt edema. Her biochemistry revealed normal renal function (eGFR 65.74 mL/(min*1.73m^2^)), normal albuminemia (4.09 g/dl), 3.4 g/d proteinuria, and no hematuria (RBC 1–3/HP). Anti-PLA2R antibodies were detected using a commercial ELISA kit (EUROIMMUN, Lübeck, Germany) and were 38 (normal range < 20) RU/ml. Her viral serology was negative for HBV, HCV, HIV, and Syphilis. The malignancies screening was negative. She had normal complement C3 and C4 levels. ANA, anti-dsDNA, anti-neutrophil cytoplasmic antibodies (ANCA), and anti-GBM tests were negative.

**Fig. 1:**
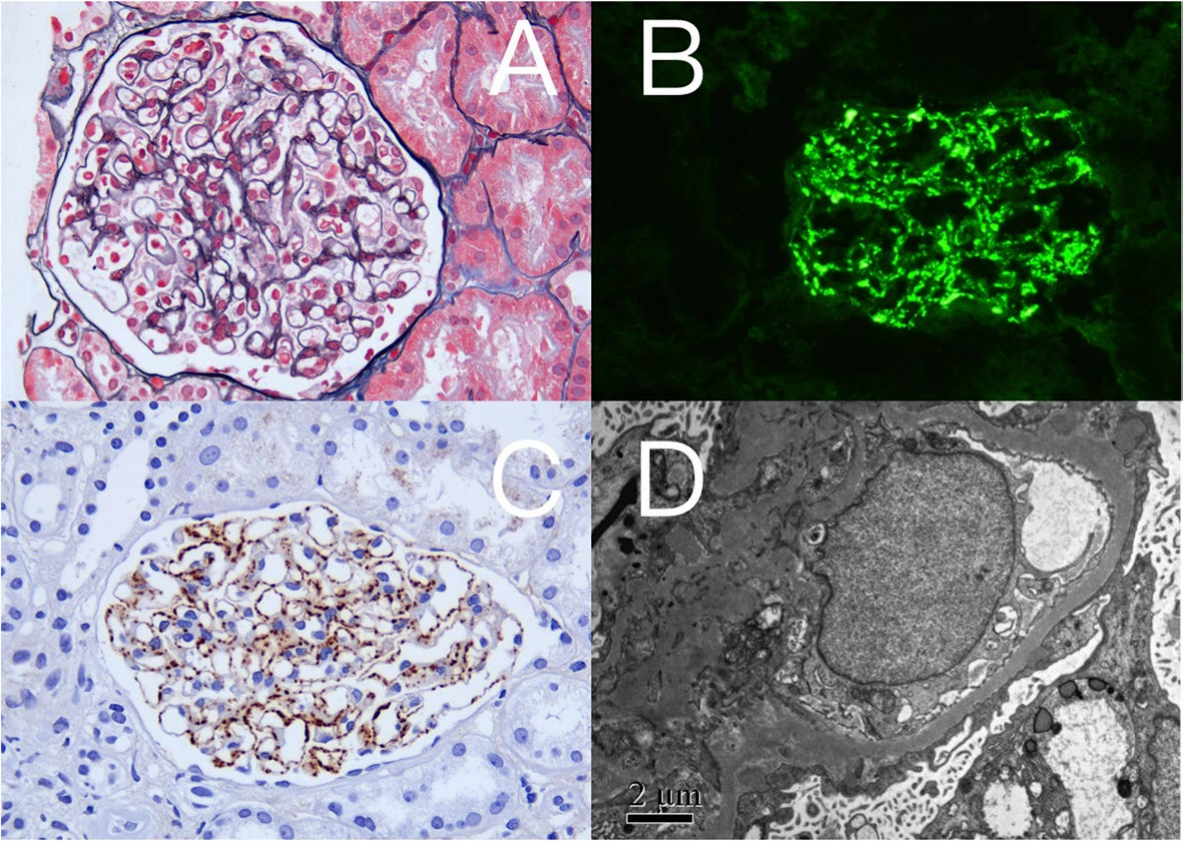
Kidney biopsy of case 1. **A** Mild thickening of glomerular basement membrane (PASM, × 400); **B** Granular IgG deposition along the glomerular basement membrane and in the mesangium (immunofluorescence, × 200); **C** Granular PLA2R staining along the glomerular capillary wall (immunohistochemistry, × 400); **D** Subepithelial electron-dense deposits under electron microscopy (× 10,000)

She received a kidney biopsy. Immunofluorescence microscopy showed granular immunoglobulin (Ig) G and C3 deposits along the glomerular basement membrane and in the mesangium, with no light chain restriction (Fig. 1B). Light microscopy showed mild thickening of the glomerular basement membrane (Fig. 1A). Immunohistochemical staining for PLA2R was +++ (Fig. 1C). Electron microscopy showed diffused podocyte foot-process effacement and numerous distributed subepithelial electron-dense deposits (Fig. 1D).

The patient was diagnosed as PLA2R-related MN and treated with irbesartan 300 mg/d, hydrochlorothiazide 25 mg/d, and spironolactone 20 mg/d. Urinary protein levels were 2.6–2.9 g/d and serum creatinine remained stable during follow-up.

### Case 2

This 52-year-old female patient is the sister of the patient above. Six months ago, she was admitted for having proteinuria for 2 months. She had hypertension for 8 years and diabetes mellitus for 3 years. She was not a smoker. Laboratory data showed normal renal function [eGFR 92.44 mL/(min*1.73m^2^)], hypoalbuminemia (3.05 g/dl), 2.55 g/d proteinuria, and urinary red blood cells at 4–6/HP. Serum samples were tested negative for anti-PLA2R antibody. Her viral serology was negative for HBV, HCV, HIV, and Syphilis. The malignancies screening was negative. She had normal complement levels and ANA, anti-dsDNA antibodies, ANCA, and anti-GBM antibodies were negative.

**Fig. 2:**
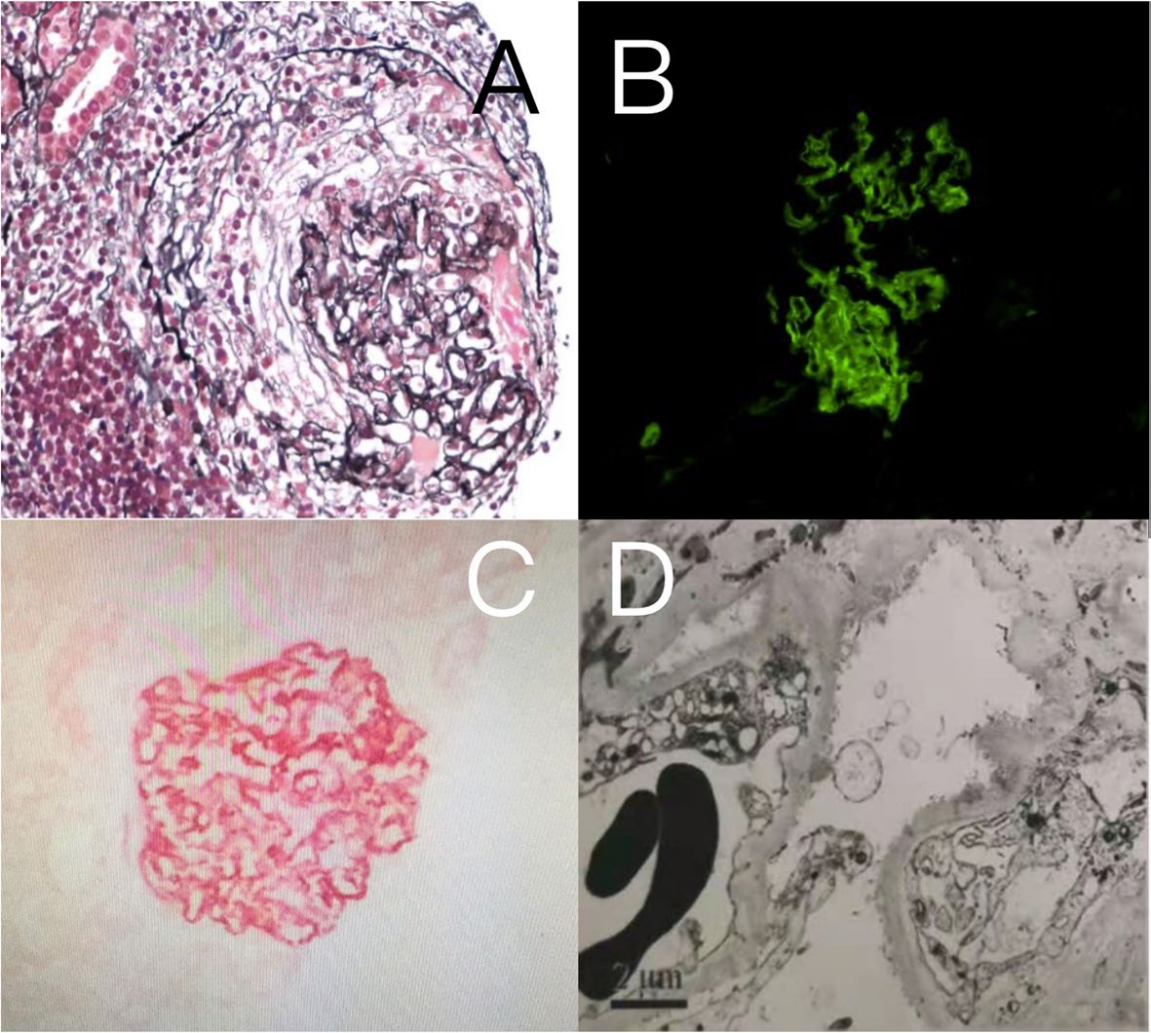
Kidney biopsy of case 2. **A** the presence of fibrocellular crescent and mild thickening of the glomerular basement membrane (PASM, × 400). **B** Granular staining of IgG along glomerular capillary loop (immunofluorescence, × 200); **C** Granular PLA2R staining along the glomerular capillary wall (immunohistochemistry, × 200); **D** Diffused podocyte foot-process detachment and subepithelial electron-dense deposits under electron microscopy (× 12,000)

A kidney biopsy was performed. Immunofluorescence showed diffuse granular staining of IgG and C3 along the glomerular capillary loop, and granular mesangial staining of IgA, IgM, and C3 (Fig. 2B). Light microscopy showed nine glomeruli, with one fibrocellular crescent. Glomerular basement membrane was mildly thickened with tiny subepithelial fuchsinophilic stains without mesangial proliferation (Fig. 2A). Immunohistochemical staining for PLA2R was positive (Fig. 2C). Electron microscopy showed diffused podocyte foot-process detachment and distributed subepithelial and mesangial electron-dense deposits (Fig. 2D). The patient has been diagnosed with PLA2R-associated MN and IgA nephropathy.

The patient was treated with empagliflozin and irbesartan. 2 months later, she presented with nausea, oliguria, and edema, but denied fever, rash, shortness of breath, hemoptysis, or gross hematuria. The physical examination showed a temperature of 36.8 °C, a blood pressure of 117/75 mmHg, and pedal edema. Her biochemistry showed acute renal failure [eGFR 3.01 mL/(min*1.73m^2^)], hypoalbuminemia (1.78 g/dl), proteinuria (1.08 g/d), and hematuria (RBC 184/HP). Anti-GBM antibody was +++ and ANCA was negative (immunoblotting test). Her hemoglobin was 8.4 g/dL. A kidney ultrasound showed an enlarged kidney. Chest X-ray was normal.

The patient refused to take a repeat kidney biopsy. Based on the clinical phenotype of rapidly progressive glomerulonephritis and the positive serology of anti-GBM antibody, the patient was diagnosed with anti-GBM disease combined with MN. She received daily plasma exchange until anti-GBM antibodies turned into negative, pulsed intravenous methylprednisolone plus cyclophosphamide. Two months later, she withdrew dialysis with Scr 3.05 mg/dl [eGFR 16.83 mL/(min*1.73m^2^)]. After 1 year, anti-GBM antibodies remained negative and Scr was stable at 2.33 mg/dl [eGFR 23.35 mL/(min*1.73m^2^)] with urinary protein less than 1 g/d.

### HLA typing

HLA Class, I and Class II (A, B, C, DRB1, DQB1, and DPB1) genes were typed using SeCoreTM Sequencing Kits (Invitrogen, Brown Deer, WI). Allele typing reports were issued using uTYPETM HLA Sequence Analysis Software, which analyzes the output data based on IMGT/HLA database to determine molecular typing.

The sibling sisters were found to have the same HLA alleles: A*02:01/*33:03, B*44:03/*55:02, C*01:02/*07:06, DQB1*02:01:01:01/*05:02:01:01, DPB1*03:01:01:01/*05:01:01:01, DRB1*03:01/*15:01.

## Discussion and conclusion

This report described two siblings who presented with non-nephrotic range proteinuria and were diagnosed with pathology-confirmed PLA2R-related MN 1 year apart. One of them developed anti-GBM disease after the onset of MN. The high-resolution HLA typing showed identical alleles in both siblings, specific heterozygote of DRB1*15:01/*03:01. Both alleles are reported as independent risk alleles for PLA2R-related MN and DRB1*15:01 is associated with anti-GBM disease [9–11].

The susceptible HLA alleles of primary MN are suggested to be DRB1*03:01 in both European and Asia, while DRB1*15:01 in Asia [8–10]. The identification and studies on familial PLA2R-related MN are expected to provide novel information on genetic susceptibility. Most reports of familial MN were published in the last century without high-resolution HLA typing and antigen identification [13–17]. There was one report having done high-resolution HLA typing only for DRB1 and DQB1 in a familial MN [17]. But only one of the twins was PLA2R-related MN [17]. To our knowledge, this was the first study using high-resolution HLA typing in siblings developing PLA2R-related MN.

DRB1*03:01 allele was reported to be susceptible for primary MN in the Chinese population, strongly associated with anti-PLA2R antibody positive patients [9] and higher levels of anti-PLA2R antibodies [18]. Another study from China found DRB3*02:02 was an independent risk allele for both PLA2R-related MN and higher levels of anti-PLA2R antibodies, which allele was strongly linked to DRB1*03:01 [10]. Our results supported that the haplotype DRB1*03:01 plays an important role in the development of PLA2R-related MN. HLA-DRB1*15:01 was another risk allele found in PLA2R-related MN in the Chinese population [10]. It has been reported consistently with the haplotypes DRB1*15:01-DQB1*06:02. The siblings carry no DQB1*06:02 and supported DRB1*15:01 as the risk allele in PLA2R-related MN.

Our second patient developed GBM disease several months after the diagnosis of MN, suggesting the possibility of conversion to GBM disease followed MN. Previous studies have reported anti-GBM disease occurring with or secondary to membranous nephropathy [6, 19]. MN secondary to GBM disease may be associated with a local immune response to MN leading to GBM abnormalities that favor stimulation of anti-GBM antibody production by α3 (IV) NC1 conformational changes. DRB1*15:01 is a risk allele for both anti-GBM disease and membranous nephropathy [8, 11], suggesting the mechanism of the combination of MN and anti-GBM disease may be associated with the same HLA susceptibility genotype. The siblings had the same HLA type and only one of them developed anti-GBM disease. Given they lived separately after childhood, this supported the contribution of the environment to disease pathogenesis.

Although, it is difficult to exclude anti-GBM disease in this patient developed secondary to IgA nephropathy. Since the patient’s kidney biopsy showed very mild pathological changes of IgA nephropathy under light microscopy, and there was no obvious clinical manifestation of nephritis syndrome. Moreover, it has been reported patients with combined MN and anti-GBM disease have higher levels of urinary protein, a lower proportion of glomerular crescents, and higher rates of kidney function recovery, after the treatments of plasma exchange combined with glucocorticoid and cyclophosphamide [6]. The clinical manifestations of case 2 were consistent with these characteristics. We prefer that the patient’s anti-GBM disease was secondary to membrane nephropathy rather than IgA nephropathy. But, indeed, we cannot exclude the possibility of secondary to IgA nephropathy.

The proposed mechanism of better kidney outcome includes a narrower antigen spectrum of anti-GBM antibodies, lower reactivity against NC1 domains, and less anti-α3(IV)NC1 IgG1 and IgG3 in these patients [6]. Early identification and intensive treatments for such patients are very important to improve the prognosis.

In summary, we describe a familial case of PLA2R-related MN sharing a strong genetic concordance, supporting the role of genetic factors that HLA-DRB1*15:01 and DRB1*03:01 predispose patients in the development of PLA2R-related MN in Han Chinese population. The combination of MN and anti-GBM disease may also partially be associated with the same susceptible HLA allele DRB1*15:01.

### Patient perspective

Regular follow-up visits allowed me to detect the problem of kidney function early and receive timely examination and treatment. I never thought that plasma replacement and glucocorticoids and other medications could help me get off dialysis, which was so lucky.

## Data Availability

All data generated or analyzed during this study are included in this published article.

## Abbreviations

pMN: Primary membranous nephropathy
PLA2R: Phospholipase A2 receptor
MN: Membranous nephropathy
HLA: Human leukocyte antigen
THSD7A: Thrombospondin type-1 domain-containing 7A
NELL-1: Neural epidermal growth factor-like 1
GBM: Glomerular basement membrane
α3(IV)NC1: The noncollagenous domain of α3 chain of type IV collagen
Scr: Serum creatinine
ANCA: Neutrophil cytoplasmic antibodies
Ig: Immunoglobulin
